# Highly-efficient three-dimensional waveguide couplers using impedance-tunable transformation optics

**DOI:** 10.1038/s41598-018-27300-9

**Published:** 2018-06-14

**Authors:** Jun Cao, Fenghua Qi, Senlin Yan, Lifa Zhang

**Affiliations:** 1 Department of Physics and Institute of Theoretical Physics, Nanjing Normal University, Nanjing, 210023 China; 2 Department of Electronics Engineering, Nanjing Xiaozhuang University, Nanjing, 211171 China

## Abstract

There is growing interest in designing waveguide couplers with low reflections. Using impedance-tunable transformation optics, we propose a coordinate transformation technique in the design of compact three-dimensional waveguide couplers. To transmit electromagnetic waves between two coaxial waveguides with different inner and outer radii in the microwave range, a suitable impedance function is derived to reduce the impedance mismatch at the boundary, which make the fabrication process being simplified due to the reduced set of transformation media(dielectric response materials only). A larger refractive index is set to raise the coupling performance in the low frequency range. Next we apply impedance-tunable transformation optics to the design of dielectric waveguide couplers, where only the core region be contained in the transformed space; by selecting a tunable impedance function, waves can transmit efficiently through waveguides with quite different cross sections and background media. The proposed impedance-tunable three-dimensional waveguide couplers are confirmed by the 3-dimensional numerical simulation with good performances, which can have potential applications in fiber-to-chip coupling.

## Introduction

Based on the invariance of Maxwell’s equations with coordinate transformations, the theory of transformation optics, was proposed by Pendry^1^ and Leonhardt^2^, which has become a powerful method in the design of optical components to manipulate electromagnetic (EM) waves^3–12^. The coordinate transformation method can also be extended in the manipulation of other types of waves, such as matter waves^13,14^, sound waves^15–17^, and has also been applied to the manipulation of heat flow^18–20^.

Electromagnetic couplers are important devices, which reduce mode mismatch and enable efficient transfer of EM waves between waveguides with different cross sections and background media. To reduce any coupling losses, couplers with different mechanisms have been proposed in the past. These include grating couplers^21–24^, parabolic reflectors^25^, and luneburg lens^26^. However, the reported coupling efficiencies with these conventional methods remain very low. In other words, efficient coupling of EM waves in waveguides is still needed. Recently gradient index metamaterials (GIMs) have been applied to the guiding of EM waves based on the mechanism of propagating waves to surface waves converting^27^, and some meaningful applications have been proposed due to its easier realization of the needed isotropic materials, such as the waveguide bends, splitters, and the different modes coupling^28^. But for coupling waves efficiently from different cross section of waveguides, especially for 3D couplers, it is still unknown whether the above method can be applied. Much effort has been made in the coupler design by using the coordinate transformation technique^29–31^. For two dimensional coupling, however, the impedance mismatch at the waveguide boundaries is unavoidable due to unequal coordinate stretching in two dimensions. As a result, reflections limit its practical applications in many cases. Three-dimensional coordinate transformation was already proposed by Emiroglu^32^ to obtain an impedance-matched compressor/expander. This approach can be applied to some special cases, where the expansion/compression rates are identical in two orthogonal directions, and the connected media are identical too. Quasi-conformal mappings (QCMs) have been presented to minimize the anisotropy of the transformation medium in many cases, which can be realized by inhomogeneous isotropic medium, and has been applied on the 2D EM coupler design^33,34^; but the method can hardly be extended to the 3D coupler design. To achieve easier realization materials, photonic crystal realization also have been proposed in the transformation-optical design of 2D coupler^35^. To remove the impedance mismatch at the boundary and obtain reduced parameter materials at the same time, a generalized theory of impedance-tunable transformation optics was proposed for the geometrical optics limit^36^, which provides more flexibilities to the controlling and guiding of EM waves, and enables us more opportunities in realizing the transformation media.

In this paper, we propose a three-dimensional coordinate transformation scheme for the design of waveguide couplers using impedance-tunable transformation optics. First we apply the scheme to the coaxial waveguide coupler design in the microwave range, where different outer/inner radii ratios can be used for different configurations. By setting suitable impedance functions to match the impedance at the boundary, highly-efficient couplers can be designed, where the reduced set of transformation media will make the fabrication process be simplified. Then we study the dielectric waveguide coupler design for coupling light efficiently from large size waveguide of low index to small size waveguide of high index, by setting appropriate impedance function the EM waves can be efficiently transmitted between different cross sections and background media.

## Methods

### Theoretical design

Within the theory of impedance-tunable transformation optics^36^, the relative permittivity ${\varepsilon }^{i^{\prime} j^{\prime} }$ and the permeability ${\mu }^{i^{\prime} j^{\prime} }$ of the transformation medium, for a given coordinate transformation *x*′ = *x*′(*x*), can be expressed as

1 $$\begin{array}{rcl}{\varepsilon }^{i^{\prime} j^{\prime} } & = & |\det ({A}_{i}^{i^{\prime} }){|}^{-1}{A}_{i}^{i^{\prime} }{A}_{j}^{j^{\prime} }\varepsilon {\delta }^{ij}/k\\ {\mu }^{i^{\prime} j^{\prime} } & = & |\det ({A}_{i}^{i^{\prime} }){|}^{-1}{A}_{i}^{i^{\prime} }{A}_{j}^{j^{\prime} }\mu k{\delta }^{ij}\end{array}$$

Here, the impedance coefficient *k* is a spatial continuous function.

In this study, we generalize the theory of impedance-tunable transformation optics to the design of waveguide couplers such that the refractive index in the original space can also be tunable. and the relative permittivity and permeability in the original space are reset to be *ε*^*ij*^ = *pδ*_*ij*_/*k* and *μ*^*ij*^ = *pkδ*_*ij*_. It means that the relative permittivity and permeability of the transformation medium are changed to be $p{\varepsilon }^{i^{\prime} j^{\prime} }$ and $p{\mu }^{i^{\prime} j^{\prime} }$. The constant refractive index *p* cannot change the light rays compared to the conventional transformation (*p* = 1). The large *p* can expand the application of the geometrical-optics approximation and improve the performance of the coupler at low frequencies.

### Numerical calculation

The numerical simulation was conducted using the software COMSOL Multiphysics.

## Results

### Coaxial waveguide coupler

**Figure 1:**
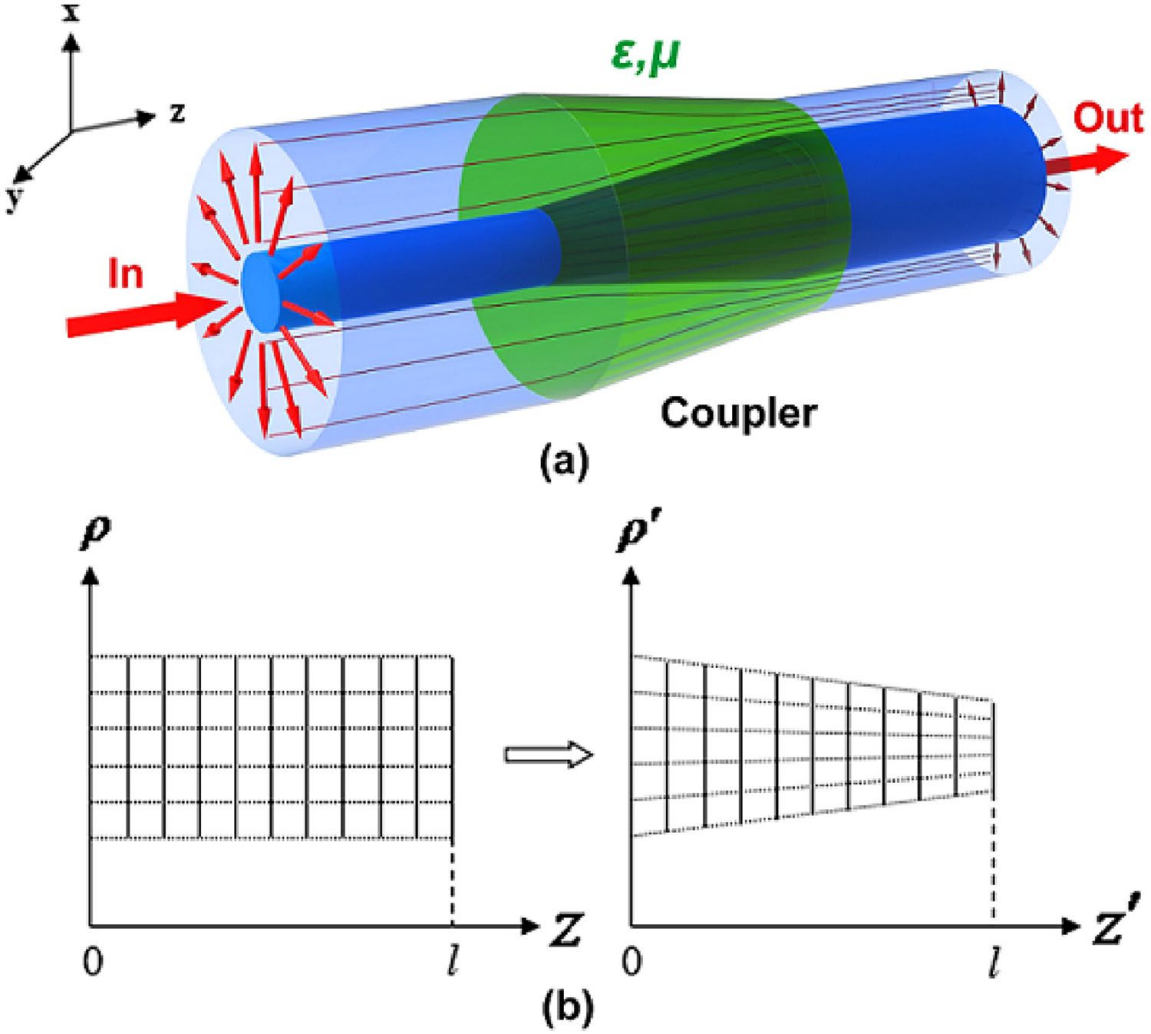
Schematics of the coaxial waveguide coupler using impedance-tunable transformation optics. (**a**) Structure, where the outer and inner radius of WG_1_ and WG_2_ are (**a**–**d**) respectively. The length of the coupler is *l*. It is embedded within a transformation medium. (**b**) Illustration of the coordinate transformation.

Using three-dimensional coordinate transformation, a coaxial waveguide coupler to connect coaxial waveguides with different outer and inner radii is designed. The proposed structure is shown in Fig. 1(a), where the waveguides WG_1_ and WG_2_ with their respective outer and inner radii *a*, *b* and *c*, *d* are connected via a linear taper structure, with the length *l*. To couple waves efficiently between WG_1_ and WG_2_ in the +*z* direction, tapers are embedded within a transformation medium, Fig. 1(b) shows the coordinate transformation between the transformed space (*ρ*′, *θ*′, *z*′) and the original space (*ρ*, *θ*, *z*). We define the following transformation:

2 $$\begin{array}{ccc}\rho ^{\prime} =\rho -\frac{\rho e+f}{l(b-a)}z, & \theta ^{\prime} =\theta , & z^{\prime} =z\end{array}$$

where *e* = *b* + *c* − *a* − *d* and *f* = *ad* − *bc*.

Based on the above equation, the Jacobian matrix for the transformation *A* = ∂(*ρ*′, *θ*′, *z*′)/∂(*ρ*, *θ*, *z*), expressed in a cylindrical (*ρ*′, *θ*′, *z*′) coordinate system, can be obtained as

3 $$A=[\begin{array}{ccc}{a}_{11} & 0 & {a}_{13}\\ 0 & {a}_{22} & 0\\ 0 & 0 & {a}_{33}\end{array}]$$

where ${a}_{11}=\frac{\partial \rho ^{\prime} }{\partial \rho }=1-\frac{ez^{\prime} }{l(b-a)}$, ${a}_{13}=\frac{\partial \rho ^{\prime} }{\partial z}=-\,\frac{\frac{\rho ^{\prime} l(b-a)+z^{\prime} f}{l(b-a)-z^{\prime} e}e+f}{l(b-a)}$, ${a}_{22}=\frac{\rho ^{\prime} \partial \theta ^{\prime} }{\rho \partial \theta }=\frac{1-\frac{z^{\prime} e}{l(b-a)}}{1+\frac{z^{\prime} f}{\rho ^{\prime} l(b-a)}}$, ${a}_{33}=\frac{\partial z^{\prime} }{\partial z}=1$, and ${\rm{\det }}\,A={a}_{11}{a}_{22}=\frac{{(1-\frac{z^{\prime} e}{l(b-a)})}^{2}}{(1+\frac{z^{\prime} f}{\rho ^{\prime} l(b-a)})}$ is the determinant value. Using standard calculations, both relative permittivity *ε* and permeability *μ* of the transformation medium can be obtained as

4 $$\varepsilon =[\begin{array}{ccc}{\varepsilon }_{\rho \rho } & 0 & {\varepsilon }_{\rho z}\\ 0 & {\varepsilon }_{\theta \theta } & 0\\ {\varepsilon }_{z\rho } & 0 & {\varepsilon }_{zz}\end{array}]\,\mu =[\begin{array}{ccc}{\mu }_{\rho \rho } & 0 & {\mu }_{\rho z}\\ 0 & {\mu }_{\theta \theta } & 0\\ {\mu }_{z\rho } & 0 & {\mu }_{zz}\end{array}]$$

where

5 $$\begin{array}{ll}{\varepsilon }_{\rho \rho }=\frac{p}{k}\frac{({a}_{11}^{2}+{a}_{13}^{2})}{\det \,A}, & {\mu }_{\rho \rho }=pk\frac{({a}_{11}^{2}+{a}_{13}^{2})}{\det \,A}\\ {\varepsilon }_{\rho z}={\varepsilon }_{z\rho }=\frac{p}{k}\frac{{a}_{13}{a}_{33}}{\det \,A}, & {\mu }_{\rho z}={\mu }_{z\rho }=pk\frac{{a}_{13}{a}_{33}}{\det \,A}\\ {\varepsilon }_{zz}=\frac{p}{k}\frac{1}{\det \,A}, & {\mu }_{zz}=pk\frac{1}{\det \,A}\\ {\varepsilon }_{\theta \theta }=\frac{p}{k}\frac{{a}_{22}^{2}}{\det \,A}, & {\mu }_{\theta \theta }=pk\frac{{a}_{22}^{2}}{\det \,A}\end{array}$$

Different incident mode waves correspond to different *k* functions if the reflections at the boundary are avoided. This requires different transformation media. In our design, we only focus on incident TEM mode. The TEM mode is the dominant mode in coaxial waveguides without cutoff frequencies. To suppress the transmission of high order modes in coaxial waveguides, i.e. only the TEM mode is allowed, the input frequency should be less than ${f}_{{\rm{\max }}}=\frac{{c}_{0}}{\pi (a+b)}$. Here, *a* and *b* are the outer and inner radii of the coaxial waveguide, *c*_0_ = 3 × 10^8^ m.s^−1^ is the EM wave velocity in vacuum. For TEM mode waves in coaxial waveguides, the electric field is oriented in the radial direction, and the magnetic field is oriented in the tangential direction. Then, only *μ*_*θθ*_, *ε*_*ρρ*_, *ε*_*ρz*_ = *ε*_*zρ*_ and *ε*_*zz*_ enter the Maxwell equations. The electric input field can be set to $\overrightarrow{E}=\frac{{E}_{0}}{\rho }{\overrightarrow{e}}_{\rho }$, while the magnetic field is $\overrightarrow{H}=\frac{{H}_{0}}{\rho }{\overrightarrow{e}}_{\theta }$. The condition for no reflection is *k* = 1 at the boundary *z*′ = 0, where the transformation is continuous. To eliminate the reflections at the boundary *z*′ = *l*, we need to calculate the reflection coefficient R of the waves at the boundary *z*′ = *l* and obtain the correct coefficient *k*. By applying the continuity of the total tangential electric and magnetic fields at the boundary *z*′ = *l*, we obtain $k=1+\frac{f}{\rho ^{\prime} (b-a)}$. To satisfy the condition for no reflection at both boundaries simultaneously, and ensure the continuity of the transformation medium, the impedance coefficient k should be a continuous spatial function. It can be formulated as

6 $$k=1+\frac{z^{\prime} f}{\rho ^{\prime} l(b-a)}$$

The impedance function *k* is not unique but it should be not so complicated that the transformation medium can be realized. The above function *k* can be chose to make the parameter for the relative permeability be *μ*_*θθ*_ = *p*, which means it is only a dielectric-response material thus can provide more opportunities to find realizable materials in the future. Note that for the special case of $\frac{a}{b}=\frac{c}{d}$ (i.e. coaxial waveguides with equal ratios between outer and inner radii), one can obtain *k* = 1, which is the standard transformation (the non-tunable impedance case for *k* = 1). Actually, the impedance-tunable transformation optics can be applied to coupling waves between arbitrary outer and inner radii to achieve high efficiency.

To measure the performance of the designed EM wave coupler, embedded within an impedance-tunable transformation medium, we conducted three-dimensional numerical simulations using COMSOL Multiphysics. In the simulations, TEM waves were coupled from WG_1_ with minimum transmission loss(the outer/inner radii ratio is 3.59) to WG_2_ with maximum power capacity(the outer/inner radii ratio is 1.65). We let the sum of the inner and outer radii of the two waveguides be identical. Without loss of general validity, the length of the coupler can be set to *l* = 0.04 m, and the outer and inner radii of WG_1_ and WG_2_ are set to *a* = 0.0359 m, *b* = 0.01, and *c* = 0.0286 m, *d* = 0.0173 m, respectively. Here, the waves couple from annular cross sections with a large outer radius and a small inner radius to annular cross section with a small outer radius but big inner radius. In addition, the working frequency of the fundamental TEM mode should be less than *f*_max_ = 2.08 GHz. Both the inner and outer boundaries of the coaxial waveguides and connected coupler are assumed perfect electric conductors (PECs).

**Figure 2:**
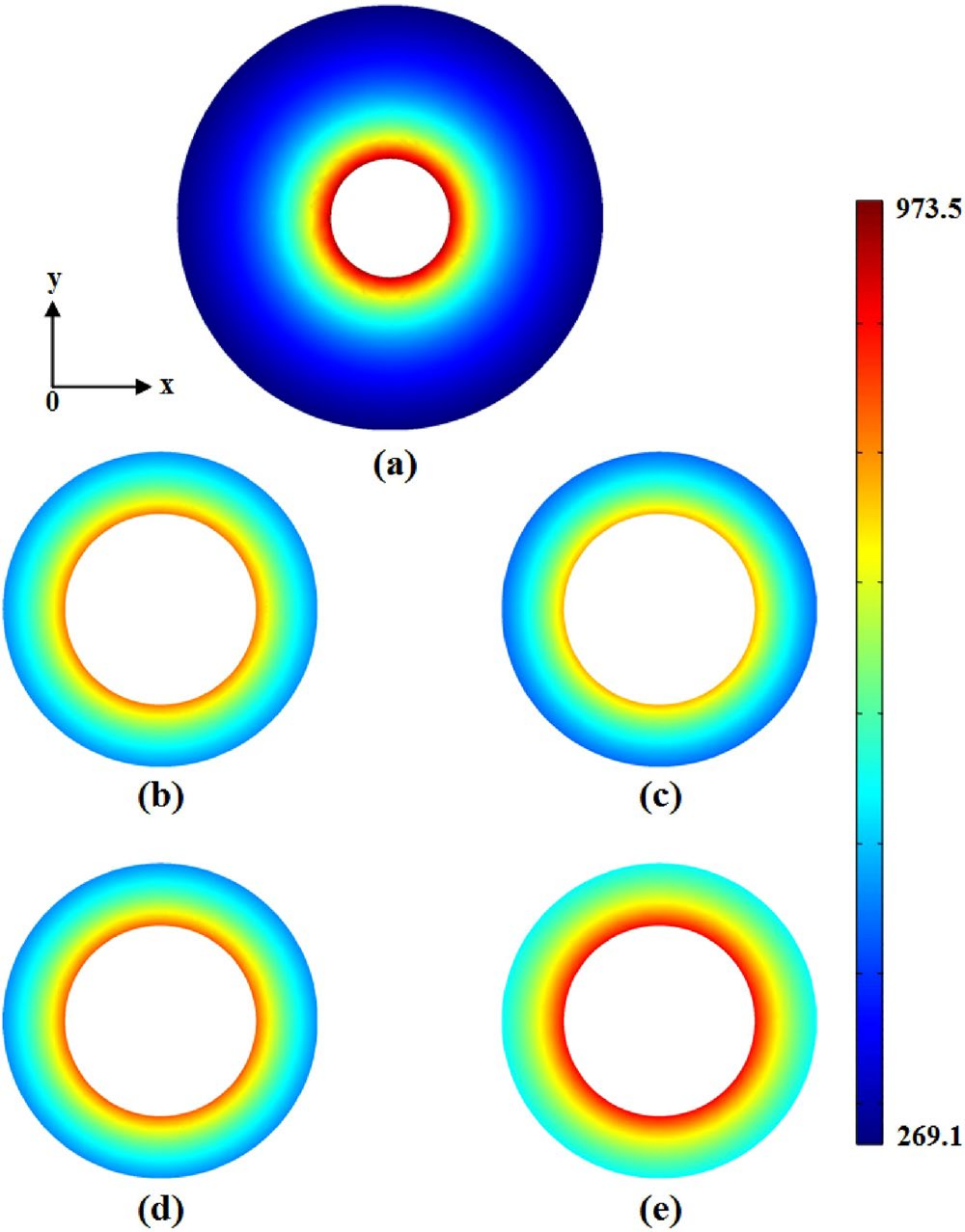
The normalized radial electric-field distribution of a coaxial waveguide-coupler. (**a**) Profile of the input-mode; (**b**) profile of the output mode i.e. the conventional approach without transformation; (**c**) output mode profile, i.e., the non-tunable impedance transformation approach; (**d** and **e**) are output mode profiles for impedance-tunable transformations with p = 1 and p = 3, respectively.

**Figure 3:**
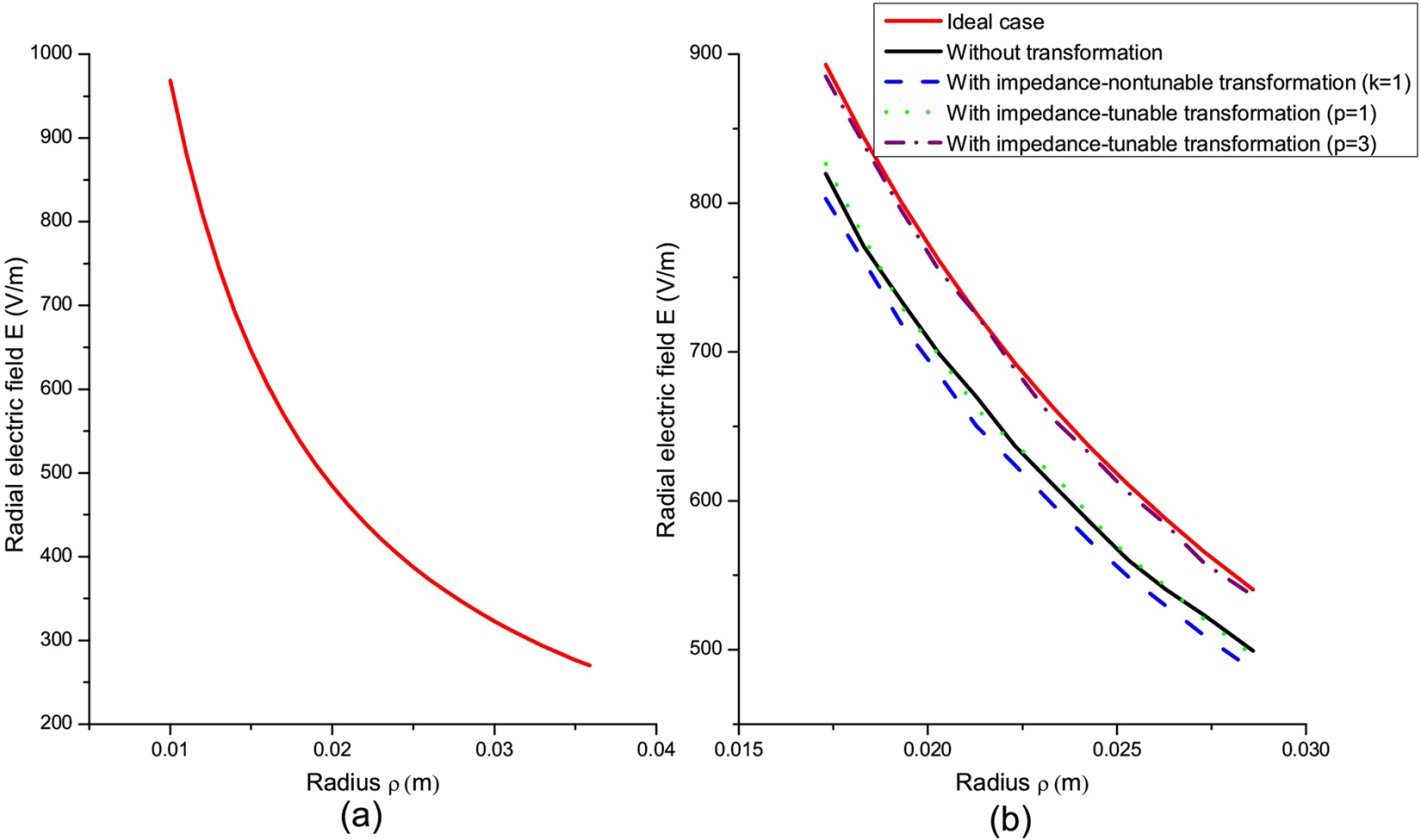
The radial electric-field intensity of the coaxial waveguide coupler. (**a**) Input, (**b**) output.

TEM mode waves are excited on the left port of WG_1_, with an incident frequency *f* = 1 GHz. Figure 2 shows the electric field simulation results, where inset (a) is the electric field of the input mode profile, and insets (b–e) relate to the output-mode profile on the right port of WG_2_. Figure 2(b) shows a conventional design example without transformation medium in the coupler. Here, the coupling efficiency *η* = *W*_2_/*W*_1_ is 84.6% due to the unavoidable reflections at the boundary. *W*_1_ and *W*_2_ are the coupler input and output powers, respectively. If a standard transformation medium is embedded in the coupler (the non-tunable impedance case for *k* = 1), even stronger reflections occur at the exit boundary– see Fig. 2(c). These take place despite good preservation of the mode profile, The resulting simulated coupling efficiency is only 80.9%. For the impedance-tunable transformation medium embedded in the coupler with *p*, the coupling efficiency increases to 85.1% - see Fig. 2(d). However, compared to direct coupling without transformation, the coupling efficiency is no higher than it due to the geometrical optics limit of the method. To overcome these shortcomings and achieve a higher coupling efficiency, a larger refractive index coefficient (*p* = 3) was selected – see Fig. 2(e). As a result, the coupling efficiency improved to 98.2%, which means there is almost no reflection. The input field value for the radial distribution curve is shown in Fig. 3(a). The calculated output field values of the radial distribution curve for the four coupling cases are shown in Fig. 3(b). Using impedance-tunable transformation optics and setting a larger refractive index, we can obtain the near ideal output field distribution with high efficiency.

**Figure 4:**
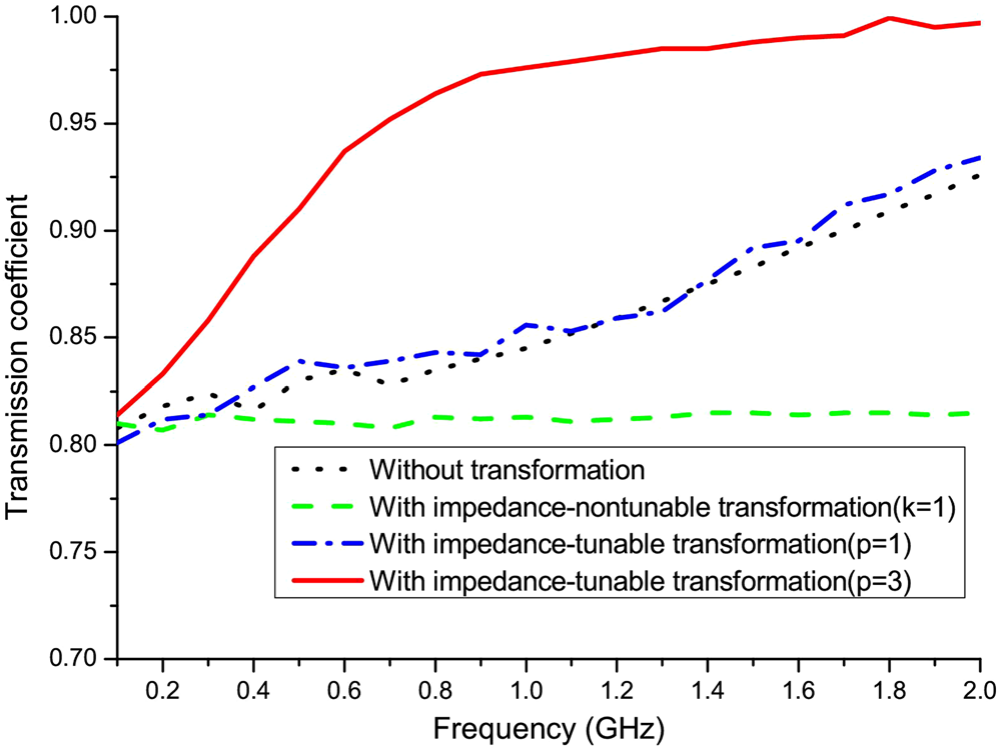
Coupling efficiency of the coaxial waveguide coupler for the TEM mode as a function of frequency.

We now investigate the coupling efficiency for a frequency range, especially for lower frequencies. Figure 4 shows the coupling efficiency of TEM modes for couplers with and without impedance-tuning, and a conventional coupler (i.e. without transformation) between 0.1 GHz to 2 GHz. the standard transformation optics does not exhibit enough competitive advantages compared to the traditional case without transformation, and thus limits its applications in many cases. By introducing a tunable impedance, the coupling efficiency can be improved. However, the coupling efficiency is still not very high, especially for the low-frequency region, and dos not demonstrate any advantage to the direct coupling without transformation. The inevitable imperfect performance due to the geometric optics limit can be improved through setting large refractive index coefficient *p*, the cost is that the relative permittivity of the transformation medium is not 1 and extreme larger parameters of *p* will increase the difficulty of realization of designed transformation medium. Nevertheless, we still can increase *p* to improve the performance at low frequencies while it is not difficult to be realized with the designed medium.

### Three-dimensional dielectric waveguide coupler

Efficient dielectric waveguide couplers are very important in optical design. Using the impedance-tunable transformation optics method, a three-dimensional compact dielectric waveguide coupler can be designed with high efficiency and less space occupation. For a dielectric waveguide, distribution of the EM field can be divided into two regions: a concentrated dielectric core and an evanescent air cladding. Therefore to couple waves with a high efficiency, the transformed space can also be divided into an inner space (major contribution) and an outer space (minor contribution). For the total transformation the embedded medium can be obtained through setting different original spaces and tuning different impedance coefficients for inner and outer parts.

**Figure 5:**
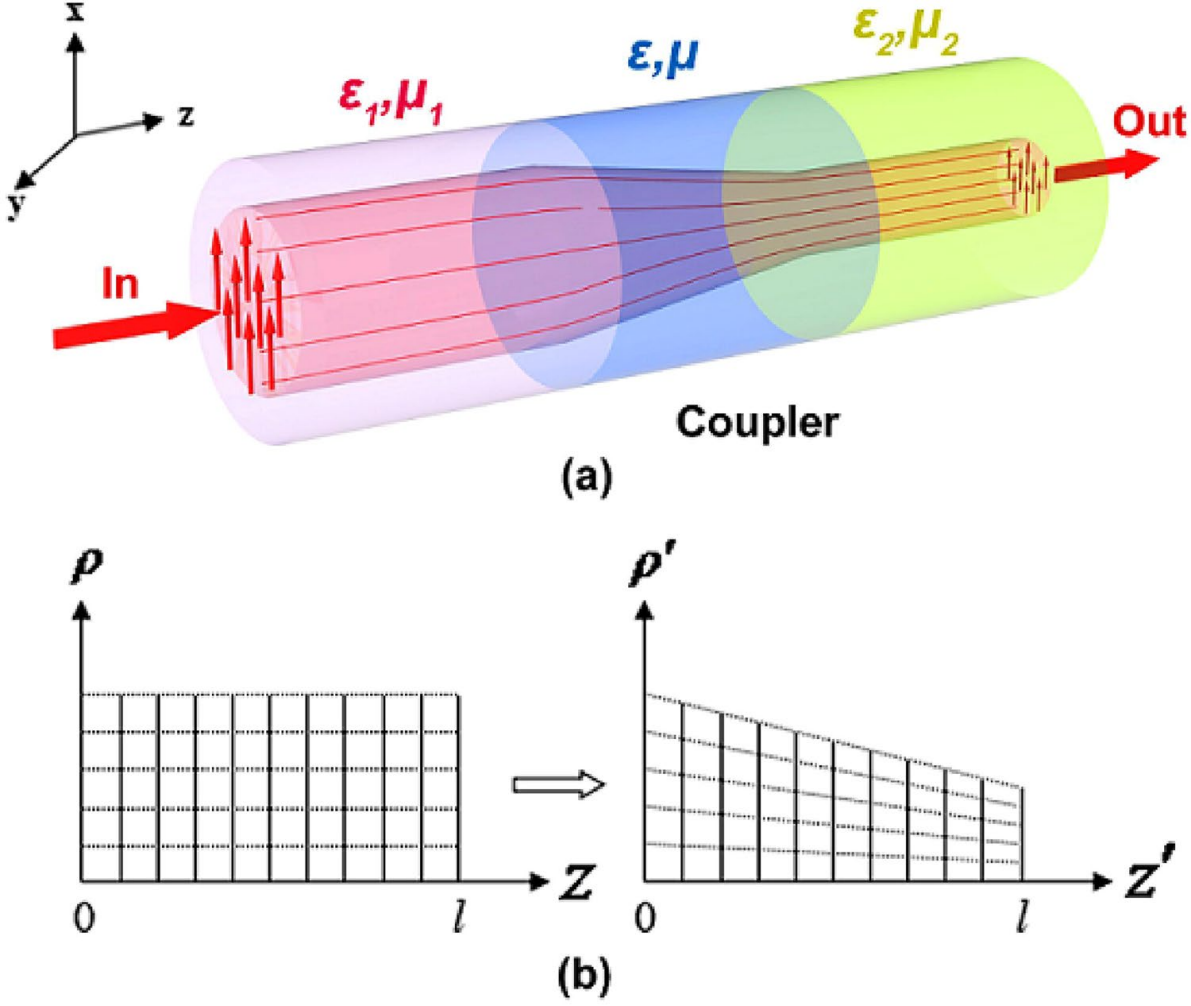
Schematic diagram of a three-dimensional dielectric waveguide coupler using impedance-tunable transformation optics. (**a**) The structure, where the relative permittivity and permeability of the core areas 1 and 2 are *ε*_1_, *μ*_1_ and *ε*_2_, *μ*_2_ respectively. The length of the coupler is *l*, and embedded within  a transformation medium. (**b**) The used coordinate transformation.

For high frequencies or in high-index-contrast waveguide, a simple method is acceptable to omit evanescent energy and only focus on the core energy coupling between different core media and cross section. A simple coupler was designed as shown in Fig. 5(a), Here, the length *l* of the coupler is connected with the core areas 1 and 2. The relative permittivity and permeability of the media in areas 1 and 2 are *ε*_1_, *μ*_1_ and *ε*_2_, *μ*_2_ respectively. To couple waves from WG_1_ to WG_2_ in the +*z* direction efficiently, the coupler is embedded within a transformation medium. Figure 5(b) shows the used coordinate transformation. The three-dimensional coordinate transformation equation can be rewritten as

7 $$\begin{array}{ccc}\rho ^{\prime} =\rho [1-\frac{z}{l}(1-\gamma )], & \theta ^{\prime} =\theta , & z^{\prime} =z\end{array}$$

where *γ* is the compression coefficient. Expansion or compression occurs, depending if *γ* is within *γ* > 1, or *γ* < 1, respectively.

Unlike the reports in ref.^32^, where the background of transformation media are the same, arbitrary background media *ε*_1_, *μ*_1_ and *ε*_2_, *μ*_2_ are discussed here. By setting the material parameters in the original space to calculate the correct impedance function *k*, we are able to remove the impedance mismatch at the boundary. The permittivity and permeability of the original space can be set to *ε*_1_/*k* and *kμ*_1_. In addition, no unique function of *k* can be selected. A normal incident plane-wave is discussed for the geometrical optics limit. Similar to the derivation in ref.^36^, the function k can be set to $1+z^{\prime} (\sqrt{\frac{{\varepsilon }_{1}{\mu }_{2}}{{\varepsilon }_{2}{\mu }_{1}}}-1)/l$.

**Figure 6:**
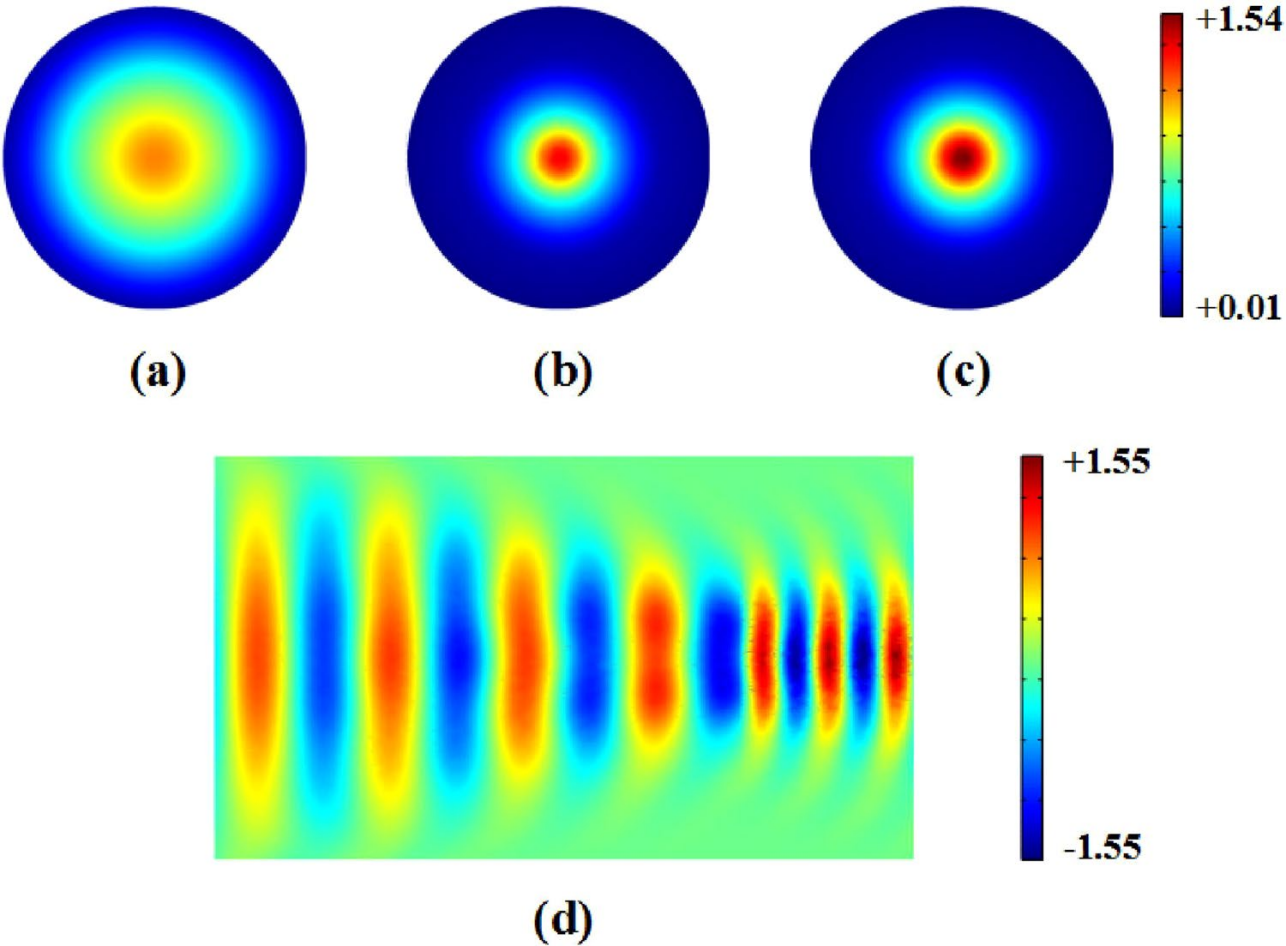
Normalized electric-field distribution (in x direction) for a 3-dimensional dielectric waveguide coupler. (**a**) Input-mode profile; (**b**) output-mode profile (for impedance non-tunable transformation); (**c**) output-mode profile (for impedance-tunable transformation); (**d**) plane with y = 0.

To investigate the performance of the EM wave coupler embedded within an impedance-tunable transformation medium, we performed three-dimensional numerical simulations using COMSOL Multiphysics for incident plane-waves. In our simulations, we used, without loss of generality, the compression coefficient *γ* = 0.5. The length of the coupler is *l* = 0.4 m, and the connected background media properties were set to *ε*_1_ = 2.25, *μ*_1_ = 1, and *ε*_2_ = 12.25, *μ*_2_ = 1 respectively. Plane waves are excited from the left of core area 1, with an incident frequency *f* = 2 GHz. Figure 6 shows the simulation results for the electric field distribution, where the inset (a) shows the electric field of the input cross section, while insets (b)-(c) show the output cross sections, and inset (d) depicts the profile for *y* = 0 plane. For a standard transformation-medium embedded in the coupler (impedance non-tunable case for *k* = 1), as shown in Fig. 6(b), despite good preservation of the wave profile, the coupling efficiency *η* is 85% due to the significant reflections. When the impedance-tunable transformation medium is embedded in the coupler, we obtain a good performance with a coupling efficiency near 98%, which means it is almost reflectionless – see Fig. 6(c). Figure 6(d) shows the total electric-field distribution in the *y* = 0 plane. The performance can also be enhanced by choosing a large refractive index coefficient *p*- similar to the design process for coaxial waveguide coupling.

## Summary

In conclusion, three-dimensional coordinate transformations was proposed for the design of highly-efficient waveguide couplers based on impedance-tunable transformation optics. By selecting the appropriate impedance function, the waves can be effectively transmitted through couplers between different cross-sections and background media, and the resulted reduced set of transformation media can provide more opportunities for fabrication in the future. A larger refractive index can be set to raise the coupling performance in the low frequency range.

## References

[1] Pendry JB, Schurig D, Smith DR (2006). Controlling Electromagnetic Fields. Science.

[2] Leonhardt U (2006). Optical Conformal Mapping. Science.

[3] Schurig D (2006). Metamaterial Electromagnetic Cloak at Microwave Frequencies. Science.

[4] Huangfu J (2008). Application of coordinate transformation in bent waveguides. J. Appl. Phys..

[5] Lai Y (2009). Illusion Optics: The Optical Transformation of an Object into Another Object. Phys. Rev. Lett..

[6] Chen H, Chan CT (2007). Transformation media that rotate electromagnetic fields. Appl. Phys. Lett..

[7] Rahm M (2008). Design of electromagnetic cloaks and concentrators using form-invariant coordinate transformations of Maxwell’s equations. Photonics Nanostruct. Fundam. Appl..

[8] Yang T (2008). Superscatterer: Enhancement of scattering with complementary media. Opt. Express.

[9] Wu YL (2016). Three-dimensional multiway power dividers based on transformation optics. Sci. Rep..

[10] Rahm M (2008). Optical Design of Reflectionless Complex Media by Finite Embedded Coordinate Transformations. Phys. Rev. Lett..

[11] Zang XF (2017). Broadband unidirectional behavior of electromagnetic waves based on transformation optics. Sci. Rep..

[12] Kim Y (2016). Designing whispering gallery modes via transformation optics. Nat. Photon..

[13] Zhang S (2008). Cloaking matter waves. Phys. Rev. Lett..

[14] Greenleaf A (2008). Approximate quantum cloaking and almost-trapped states. Phys. Rev. Lett..

[15] Cummer SA, Schurig D (2007). One path to acoustic cloaking. New. J. Phys..

[16] Chen H, Chan CT (2007). Acoustic cloaking in three dimensions using acoustic metamaterials. Appl. Phys. Lett..

[17] Zigoneanu L, Popa B, Cummer SA (2014). Three-dimensional broadband omnidirectional acoustic ground cloak. Nat. mater..

[18] Schittny R (2013). Experiments on transformation thermodynamics: molding the flow of heat. Phys. Rev. Lett..

[19] Li Y (2015). Temperature-Dependent Transformation Thermotics: From Switchable Thermal Cloaks to Macroscopic Thermal Diodes. Phys. Rev. Lett..

[20] Sklan SR (2016). Detecting Thermal Cloaks via Transient Effects. Sci. Rep..

[21] Taillaert D (2006). Grating Couplers for Coupling between Optical Fibers and Nanophotonic Waveguides. Jpn. J. Appl. Phys..

[22] Xiao Z (2013). Bandwidth analysis of waveguide grating coupler. Opt. Express.

[23] Covey J, Chen RT (2013). Efficient perfectly vertical fiber-to-chip grating coupler for silicon horizontal multiple slot waveguides. Opt. Express.

[24] Liu L (2010). High-efficiency, large-bandwidth silicon-on-insulator grating coupler based on a fully-etched photonic crystal structure. Appl. Phys. Lett..

[25] Dillon T (2008). Fiber-to-waveguide coupler based on the parabolic reflector. Opt. Lett.

[26] Arigong B (2013). Design of wide-angle broadband Luneburg lens based optical couplers for plasmonic slot nano-waveguides. J. Appl. Phys..

[27] Fu YY, Xu YD, Chen HY (2015). Applications of gradient index metamaterials in waveguides. Sci. Rep..

[28] Wang HX (2016). Broadband mode conversion via gradient index metamaterials. Sci. Rep..

[29] Tichit PH, Burokur SN, Lustrac AD (2010). Waveguide taper engineering using coordinate transformation technology. Opt. Express.

[30] Ghasemi R (2010). Efficient control of a 3D optical mode using a thin sheet of transformation optical medium. Opt. Express.

[31] Chen K (2014). Design of an ultra-short coupler in an asymmetric twin-waveguide structure using transformation optics. Appl. Opt.

[32] Emiroglu CD, Kwon DH (2010). Impedance-matched three-dimensional beam expander and compressor designs via transformation optics. J. Appl. Phys..

[33] Wu Q, Turpin JP, Werner DH (2012). Integrated photonic systems based on transformation optics enabled gradient index devices. Light Sci. Appl.

[34] Markov P, Valentine JG, Weiss SM (2012). Fiber-to-chip coupler designed using an optical transformation. Opt. Express.

[35] Liang Z, Li JS (2011). Scaling two- dimensional photonic crystals for transformation optics. Opt. Express.

[36] Cao J (2014). Reflectionless design of optical elements using impedance-tunable transformation optics. Appl. Phys. Lett..

